# Targeting histone demethylases JMJD3 and UTX: selenium as a potential therapeutic agent for cervical cancer

**DOI:** 10.1186/s13148-024-01665-3

**Published:** 2024-04-04

**Authors:** Dezhi Chen, Bo Cai, Yingying Zhu, Yimin Ma, Xiaoting Yu, Jieqi Xiong, Jiaying Shen, Weiwei Tie, Yisheng Zhang, Fei Guo

**Affiliations:** 1 Ningbo Institute of Innovation for Combined Medicine and Engineering (NIIME), Ningbo Medical Center Lihuili Hospital, Ningbo University, Ningbo, 315100 Zhejiang Province China; 2 The First Affiliated Hospital, Jiangxi Medical College, Nanchang University, Nanchang, 330006 Jiangxi Province China; 3 Jiangxi Maternal and Child Health Hospital, Nanchang, 330008 Jiangxi Province China

**Keywords:** Selenium, Cervical cancer, JMJD3, UTX, H3K27 methylation

## Abstract

**Background:**

The intriguing connection between selenium and cancer resembles a captivating puzzle that keeps researchers engaged and curious. While selenium has shown promise in reducing cancer risks through supplementation, its interaction with epigenetics in cervical cancer remains a fascinating yet largely unexplored realm. Unraveling the intricacies of selenium's role and its interaction with epigenetic factors could unlock valuable insights in the battle against this complex disease.

**Result:**

Selenium has shown remarkable inhibitory effects on cervical cancer cells in various ways. In in vitro studies, it effectively inhibits the proliferation, migration, and invasion of cervical cancer cells, while promoting apoptosis. Selenium also demonstrates significant inhibitory effects on human cervical cancer-derived organoids. Furthermore, in an in vivo study, the administration of selenium dioxide solution effectively suppresses the growth of cervical cancer tumors in mice. One of the mechanisms behind selenium's inhibitory effects is its ability to inhibit histone demethylases, specifically JMJD3 and UTX. This inhibition is observed both in vitro and in vivo. Notably, when JMJD3 and UTX are inhibited with GSK-J4, similar biological effects are observed in both in vitro and in vivo models, effectively inhibiting organoid models derived from cervical cancer patients. Inhibiting JMJD3 and UTX also induces G2/M phase arrest, promotes cellular apoptosis, and reverses epithelial-mesenchymal transition (EMT). ChIP-qPCR analysis confirms that JMJD3 and UTX inhibition increases the recruitment of a specific histone modification, H3K27me3, to the transcription start sites (TSS) of target genes in cervical cancer cells (HeLa and SiHa cells). Furthermore, the expressions of JMJD3 and UTX are found to be significantly higher in cervical cancer tissues compared to adjacent normal cervical tissues, suggesting their potential as therapeutic targets.

**Conclusions:**

Our study highlights the significant inhibitory effects of selenium on the growth, migration, and invasion of cervical cancer cells, promoting apoptosis and displaying promising potential as a therapeutic agent. We identified the histone demethylases JMJD3 and UTX as specific targets of selenium, and their inhibition replicates the observed effects on cancer cell behavior. These findings suggest that JMJD3 and UTX could be valuable targets for selenium-based treatments of cervical cancer.

**Supplementary Information:**

The online version contains supplementary material available at 10.1186/s13148-024-01665-3.

## Introduction

Cervical cancer remains a significant health challenge globally, with a pronounced impact in developing and underprivileged areas [1]. Disturbingly, global cancer statistics from 2022 reveal over 662,301 new cases and approximately 348,874 deaths attributed to cervical cancer. Within China, the incidence of cervical cancer ranks second highest globally, with more than 150,659 new cases reported in 2022 [2]. Despite the widespread availability of HPV vaccination in recent years, women beyond the eligible age for vaccination remain at an elevated risk of developing cervical cancer over the next decade or two. Current treatment modalities, including surgery, radiotherapy, and often fall short in terms of efficacy and accessibility, characterized by a low response rate (48%) and short median survival (17.51 months) [3]. Therefore, exploring innovative and cost-effective therapeutic strategies is critical.

The development of cervical cancer is closely linked with chromatin modification, which exerts a crucial influence on gene expression and plays a pivotal role in the initiation and progression of tumors [4, 5]. Among the various chromatin modifications, histone methylation on lysine residues has garnered significant attention and investigation [6, 7]. Our previous research demonstrated a significant correlation between the expression level of KDM6B and the occurrence, development, pathological grading, staging, and prognosis of various tumors [8]. Mounting evidence indicates that dysregulation of H3K27 methylation contributes to tumorigenicity [9, 10]. Studies have identified histone lysine-specific demethylases 6A (KDM6A/UTX) and 6B (KDM6B/JMJD3) as members of the KDM6 family of histone H3 lysine 27 (H3K27) demethylases [11, 12]. These demethylases promote gene activation by demethylating H3K27me3 to H3K27me2/1. H3K27me3 is associated with transcriptional silencing by altering the chromatin's physical state and impeding the access of transcription factors to enhancer and promoter regions [13–15].

Selenium is an essential trace element in the human body, mainly existing in proteins in the form of selenocysteine [16]. It plays a crucial role in maintaining organismal homeostasis. Normal plasma selenium concentrations range from 80 to 120 µg/L, and serum selenium levels below 80 µg/L are associated with an elevated risk of various cancers, including prostate, breast, stomach, and colon cancer [17, 18]. The anticancer effects of selenium have been observed at supranutritional doses of 250–300 µg/day [19]. In in vitro cell experiments, selenium has been shown to induce apoptosis through different pathways. For example, selenite induces cell cycle arrest and promotes apoptosis by altering microtubule assembly in HL60 leukemia cells [20]. Sodium selenate induces apoptosis in renal cell carcinoma through the ROS-mediated NF-κB signaling pathway [21]. Notably, selenium has been implicated in the modification of epigenetic marks [22]. However, the epigenetic inheritance of selenium in cervical cancer remains rare.

In this study, we analyzed the impact of selenium on H3K27 methylation modification to understand how selenium regulates the expression of genes related to malignant behavior in cervical cancer cells and further confirmed the anti-cervical cancer effects of selenium using organoid techniques. Overall, our findings underscore the intricate relationship between selenium, chromatin modification, and cervical cancer. Targeting histone demethylases JMJD3 and UTX could serve as a promising therapeutic strategy for the treatment of cervical cancer.

## Materials and methods

### Cell lines, culture media, and reagents for treatment

HeLa (C01-CA) human cervical adenocarcinoma cells and SiHa (C01-FH) human squamous carcinoma cells were obtained from Novobio (Shanghai, China) and cultured in DMEM and RPMI-1640 medium containing 10% fetal bovine serum (FBS, EVERY GREEN, Zhejiang, China), respectively. The cells were then incubated in a humidified atmosphere at 37 °C with 5% CO_2_. Selenium dioxide (Aldrich-200107) and GSK-J4 Hcl (S7070, a specific inhibitor of JMJD3/UTX), were purchased from Selleck (Houston, USA).

### Cell viability assay

The pharmacological effects of SeO_2_ and GSK-J4 on cell proliferation were assessed using Cell Counting Kit-8 (CCK-8, TransGen, Beijing, China) following the manufacturer's instructions. Briefly, HeLa and SiHa cells were seeded at a density of 3 × 10^3^ cells/well in 96-well culture plates and treated with varying concentrations of SeO_2_ and GSK-J4. After treatment, 20 μl of CCK-8 solution was added to each well and incubated for an additional 2 h at 37 °C in a 5% CO_2_ incubator. The optical density (OD) of each well was measured at 450 nm using a microplate reader (BioTek, VT, USA), and the data was graphically displayed. The growth rate (%) was calculated using the formula: (OD_Experiment_ − OD_Blank_)/(OD_Control_ − OD_Blank_) × 100%. The IC50 was determined using GraphPad Prism 9 software (GraphPad Software, USA).

### Colony formation assay

Cells were seeded in 6-well plates at a cell density of 1000 cells/mL or 500 cells/mL, respectively. After 48 h of culture in normal medium, the culture medium was replaced with SeO_2_/GSK-J4 or a solvent control of PBS/DMSO, and the medium was changed every 3 days. After 7 days, the cells were washed with PBS, fixed with 4% paraformaldehyde, and stained with 0.1% crystal violet solution. Colonies were counted using ImageJ software [23].

### Flow cytometry assay

To assess the cell cycle distribution and cellular apoptosis, 7 × 10^4^ cells were seeded into a 6-well plate and treated with SeO_2_ and GSK-J4. The samples for the cell cycle analysis were stained with propidium iodide (BD Biosciences, MA, USA), and the samples for the apoptosis analysis were stained with Annexin V-fluorescein isothiocyanate and propidium iodide (TransGen, Beijing, China). Flow cytometric analysis was performed using a BD FACSCanto II™ flow cytometer (BD Biosciences, MA, USA). The cell cycle distribution was analyzed with ModFit LT V3.1 (Verity Software House, USA) software and the apoptotic rate of cells was evaluated with FlowJo V10.0.7 (Treestar Inc., Ashland, OR, USA) software.

### Scratch wound healing assay

Cells were seeded onto 24-well plates and treated with SeO_2_ and GSK-J4 or a solvent control of PBS/DMSO. Prior to treatment, a clean line was created using a sterile 200 μl pipette tip. The migration of cells was observed under an inverted microscope (Leica DMI6000B, GER) and imaged at 0 h and 48 h. The relative scratch area was calculated using ImageJ software. Wound healing percentage (%) was calculated using the formula: 1 − (*S*_48h_/*S*_0h_) × 100%, where *S*_0h_ represents the initial scratch area at 0 h, and *S*_48_ h represents the scratch area at 48 h.

### Transwell chamber assay

Transwell chambers (Corning, USA) with polycarbonate membranes (8.0 μm pore size) were inserted into a 24-well culture plate. For the migration assay, HeLa cells (5 × 10^4^/well) and SiHa cells (3 × 10^4^/well) were plated into the upper chambers with 200 μl FBS-free medium containing SeO_2_ and GSK-J4 or a solvent control of PBS/DMSO. For the invasion assay, the upper chamber was evenly coated with 100 μl of Matrigel (Corning, USA) and an FBS-free medium mixture (1:8), and the remaining steps were analogous to those of the cell migration tests. Next, 800 μl of complete medium (10% FBS) was added to the lower chamber as a chemoattractant. After incubation at 37 °C in 5% CO_2_, the cells on the upper surface of the filters were removed with a cotton swab, and the invading cells on the lower surface were fixed with 4% paraformaldehyde for 30 min and stained with a 0.1% crystal violet solution for 30 min. The migration and invasion cells on the lower surface of the membrane in each chamber were counted randomly under high power fields.

### RNA extraction, reverse transcription, and quantitative real-time PCR

After treatment with either solvent control PBS/DMSO or SeO_2_/GSK-J4 for different time periods, total RNA was extracted from cells using RNAiso Plus Reagent (Takara, Japan) in accordance with the manufacturer's instructions. Next, 1 μg of total RNA was reverse transcribed into cDNA using a reverse transcriptase Kit (TransGen, Beijing, China). The mRNA expression was determined using real-time PCR with the PerfectStartTM Green qPCR SuperMix Kit (TransGen, Beijing, China) on a StepOne Plus PCR system (Applied Biosystems, Waltham, MA, USA). The 2^−ΔΔCT^ formula was used to calculate the expression of the genes, with the human glyceraldehyde-3-phosphate dehydrogenase (GAPDH) gene serving as the internal reference. The primer information is listed in Additional file 1: Table S1.

### Western blotting

Whole-cell lysates were obtained from cells treated with PBS/DMSO or SeO_2_/GSK-J4 using RIPA lysis buffer supplemented with a protease inhibitor cocktail. The lysates were then sonicated and centrifuged, and the supernatant was mixed with loading buffer and heated. Equal amounts of protein samples were separated by SDS-PAGE and transferred to PVDF membranes. After blocking with 5% skim milk, the membranes were incubated with primary antibodies overnight at 4 °C, followed by secondary antibodies and detection using an enhanced chemiluminescence (ECL) kit. Antibodies against KDM6B/JMJD3, H3K27me1, Bax, Bcl-2, Caspase-7, KDM6A/UTX, H3K27me3, Cyclin B1, Caspase-3, E-cadherin, N-cadherin, Vimentin, GAPDH, β-Tubulin, MMP-1, MMP-2, MMP-9, and CDK1 were used. The blot signals were detected using an Ultrasensitive ECL Detection Kit (Proteintech, Wuhan, China) and visualized by the ImageQuant LAS 500 (Cytiva, USA).

The antibodies used were: KDM6B/JMJD3 (1:1000, ab169197), H3K27me1 (1:500, ab194688), Bax (1:500, ab53154), Bcl-2 (1:1000, ab196495), and Caspase-7 (1:200, ab25900) from Abcam (Cambridge, UK); KDM6A/UTX (1:500, PA5-31828) from Invitrogen-Thermo Fisher Scientific (CA, USA); H3 (1:2000, #4499), H3K27me3 (1:1000, #9733), Cyclin B1 (1:1000, #12231), Caspase-3 (1:1000, #9662S), E-cadherin (1:1000, #3195), N-cadherin (1:1000, #13116), Vimentin (1:1000, #5741), GAPDH (1:1000, #2118), and β-Tubulin (1:1000, #2128) from Cell Signaling Technology (MA, USA); and MMP-1 (1:500, #10371-2-AP), MMP-2 (1:500, #10373-2-AP), MMP-9 (1:500, #10375-2-AP), CDK1 (1:500, #19532-1-AP), and horse radish peroxidase (HRP)-conjugated goat secondary antibodies against rabbit and mouse from Proteintech (Wuhan, China).

### Immunofluorescence staining

Cells were plated on glass coverslips in 24-well culture plates and treated with SeO_2_ and GSK-J4 or PBS/DMSO. After removing the cell supernatant, the cells were washed twice with cold PBS and fixed in 4% paraformaldehyde at 4 °C for 15 min. They were then permeabilized with 0.5% Triton X-100 at room temperature for 10 min and blocked with 2% BSA at 37 °C for 30 min. The coverslips were incubated with primary antibodies against Ki67 (1:400, Cell Signaling Technology, #9129, USA) and H3K27me3 (1:800, Cell Signaling Technology, #9733S, USA) overnight at 4 °C. After incubation with Alexa Fluor 488-conjugated secondary antibody (1:500, Abcam, ab150077, UK) at 37 °C for 1 h in the dark, the nuclei were stained with DAPI for 20 min at room temperature and washed with PBS. Staining was observed with a Confocal Laser Scanning Microscopy (CLSM, FV3000, Olympus, Tokyo, Japan), and the fluorescence intensity was analyzed by OLYMPUS cellSens 1.16 software.

### Chromatin immunoprecipitation (ChIP) assay

For the ChIP assay, the Simple ChIP Plus Enzymatic Chromatin IP Kit (Cell Signaling Technology, #9005, USA) was used according to the manufacturer's instructions. Cells were cross-linked using formaldehyde and then incubated with glycine. Chromatin was digested with Micrococcal Nuclease, and the nuclei were lysed using a VCX130 Sonicator. The chromatin-protein complexes were immunoprecipitated using anti-H3K27me3 (1:50, Cell Signaling Technology, #9733, USA) antibody, with normal rabbit IgG antibody (Cell Signaling Technology, #2729, USA) serving as the negative control. Purified DNA fragments were used as templates for RT-qPCR with primers listed in Additional file 1: Table S2.

### The generation, identification, and treatment of human cervical cancer-derived organoids (HCCOs)

HCCOs were generated using tissue samples obtained from the Obstetrics and Gynecology ward at Ningbo Medical Center, Lihuili Hospital. These samples were collected from surgical patients who had not undergone preoperative radiotherapy or chemotherapy. The tissue specimens were divided into three parts: one for paraffin embedding, one for cryopreservation in liquid nitrogen, and one for organoid construction. The tumor tissue was minced and digested with collagenase for 30 min. The resulting tissue clusters were then filtered through a 100 μm filter and embedded in a matrix called Matrigel (Corning, 356231, USA). Each well containing the embedded tissue clusters was supplemented with cervical cancer organoid medium, which was changed every 3–4 days. The medium used for culturing the HCCO was obtained from Absin (Shanghai, abs9590, China). The HCCO culture medium composition included DMEM/F12 supplemented with HEPES, N2, B27, EGF, Wnt3a, Primocin, Glutamax, Gentamicin/amphotericin B, Noggin, R-spondin 1, E2, Recombinant human A83-01, Y-27632 HCL, human FGF-10, and human FGF-β. After seeding the organoids in 24-well culture plates and amplifying them, HCCOs and the original tissues were embedded in paraffin and subjected to Hematoxylin and Eosin (HE) staining. Following that, Short Tandem Repeat (STR) DNA sequences were performed on both patient tumors and organoids to confirm the identities of the organoids. Subsequently, the HCCOs were treated with selenium or GSK-J4 at the indicated concentrations, and the growth status of the organoids was monitored. On the third day, the activity of the HCCOs derived from squamous cell carcinoma was assessed using the CCK-8 reagent, following the same methodology employed in the cell experiments. The activity of HCCOs from adenocarcinoma was assessed through conducting viable and non-viable cells staining using a Calcein AM/PI Cell Live/Dead Assay Kit (Beyotime, C2015S, China) according to the instruction from the manufacture.

### Establishment of three-dimensional bio-printed cervical cancer models and in vitro culture

According to Xie et al. [24], specimens were well minced and washed twice with DMEM/F12 (GIBCO) plus 1% penicillin/streptomycin (Sigma), 1% GlutaMAX (GIBCO), and 10-mM HEPES (GIBCO). The minced tissue was then digested into a single-cell suspension by incubating with EBSS (HyClone) containing 2.5 mg/ml collagenase D, (Roche) and 0.1 mg/ml DNaseI(Sigma) for 2–6 h and the digestion was stopped by adding cold advanced DMEM/F12. The cell suspension was then filtered through a 70 μm nylon cell strainer, and the yield cells were collected and resuspended into the culture medium into a concentration of 6.0 × 10^6^/ml.

To formulate the bioink for printing, the cell suspension was mixed up with buffer solution containing 5% gelatin (Sigma) and 1% sodium alginate (Sigma), resulting in a final cell concentration of 3.0 × 10^6^/ml. The bioink was drawn into a 1 ml syringe with a 23-gauge needle and printed using the 3D Cell Printer by SUNP Co. The models were printed in layers, with each model being a cube measuring 10 mm × 10 mm × 1.2 mm and consisting of four layers of bioink. The bioink contained a precise cell number of 2.5 × 10^5^. The shape of the HCCO models was designed as a square grid with independent holes between the frames to maximize the contact area between the cells and the culture medium. The models were collected using twelve-well plates and were used for viability tests. After printing, the HCCO models were fixed with a calcium chloride (CaCl2) solution for 1 min to provide better strength. Then, the models were supplemented with 2–3 ml of the HCCO culture medium. The culture medium was changed three times a week, and each time the models were fixed with calcium chloride.

### Subcutaneous xenograft model

Female BALB/C nude mice were obtained from Hunan Silaikejingda Laboratories (Hunan, China) for the xenograft mouse model. The mice were allowed to acclimatize to the laboratory environment for 1 week prior to the experiments and provided with ad libitum access to food and water. To establish BALB/c nude mice bearing tumors, SiHa cells (5 × 10^6^) were inoculated into the right flank of the mice in 200 μl volumes per site. On the seventh day after the injection of tumor cells, the mice were randomly divided into two groups: the sham-treated group and the SeO_2_/GSK-J4 treated group. The mice in the SeO_2_/GSK-J4 treated group received intraperitoneal injections of SeO_2_ (3 mg/kg) or GSK-J4 (50 mg/kg), while the sham-treated group received an equal volume of PBS/DMSO. These injections were administered every 2 days. Tumor length (*L*) and width (*W*) were measured using calipers every 2 days, and tumor volumes were calculated using the equation (*L* × *W*^2^)/2. After 18 days of treatment, the mice were euthanized humanely by cervical dislocation, and all tumors were collected for further analysis.

### Paraffin-embedded cervical tissue specimens and Immunohistochemical stain

We gathered paraffin-embedded cervical tissue specimens from the Department of Pathology archives of the First Affiliated Hospital of Nanchang University for JMJD3/UTX immunohistochemistry assays (IHC). The specimens included normal cervical tissues (*n* = 4), cervical squamous cell carcinoma (SqCa, *n* = 14), and endocervical adenocarcinoma (AdCa, *n* = 4). The pathology team at the hospital identified the tumor and normal tissues from the hysterectomy samples. Carcinoma samples were obtained from patients who were classified as stage I B to II A according to the 2009 International Federation of Gynecology and Obstetrics (FIGO) classification. None of the patients had undergone neoadjuvant chemotherapy or radiotherapy prior to radical operation.

Immunohistochemistry was utilized to evaluate the expression of KDM6A and KDM6B proteins in paraffin-embedded cervical tissue samples. The samples were deparaffinized and rehydrated, followed by antigen retrieval. Slides were then incubated with primary antibodies against UTX/KDM6A (1:500) and JMJD3/KDM6B (1:200), followed by secondary antibodies and staining with DAB. Positive staining appeared as brown staining in the cell membrane or nucleus. Image quantification was performed using ImageJ software.

### Statistical analysis

Data are presented as the mean ± standard Error (SEM), and all graphs and statistical analyses were conducted with GraphPad Prism 9. Statistical difference between two groups were compared using unpaired/paired Student’s *t*-test or one-way ANOVA. A value of* p* < 0.05 was considered statistically significant (*p < 0.05, **p < 0.01, ***p < 0.001, ****p < 0.0001).

## Results

### Selenium exhibits significant inhibitory effects on the proliferation, migration, and invasion of cervical cancer cells, while simultaneously promoting cell apoptosis in vitro

**Fig. 1:**
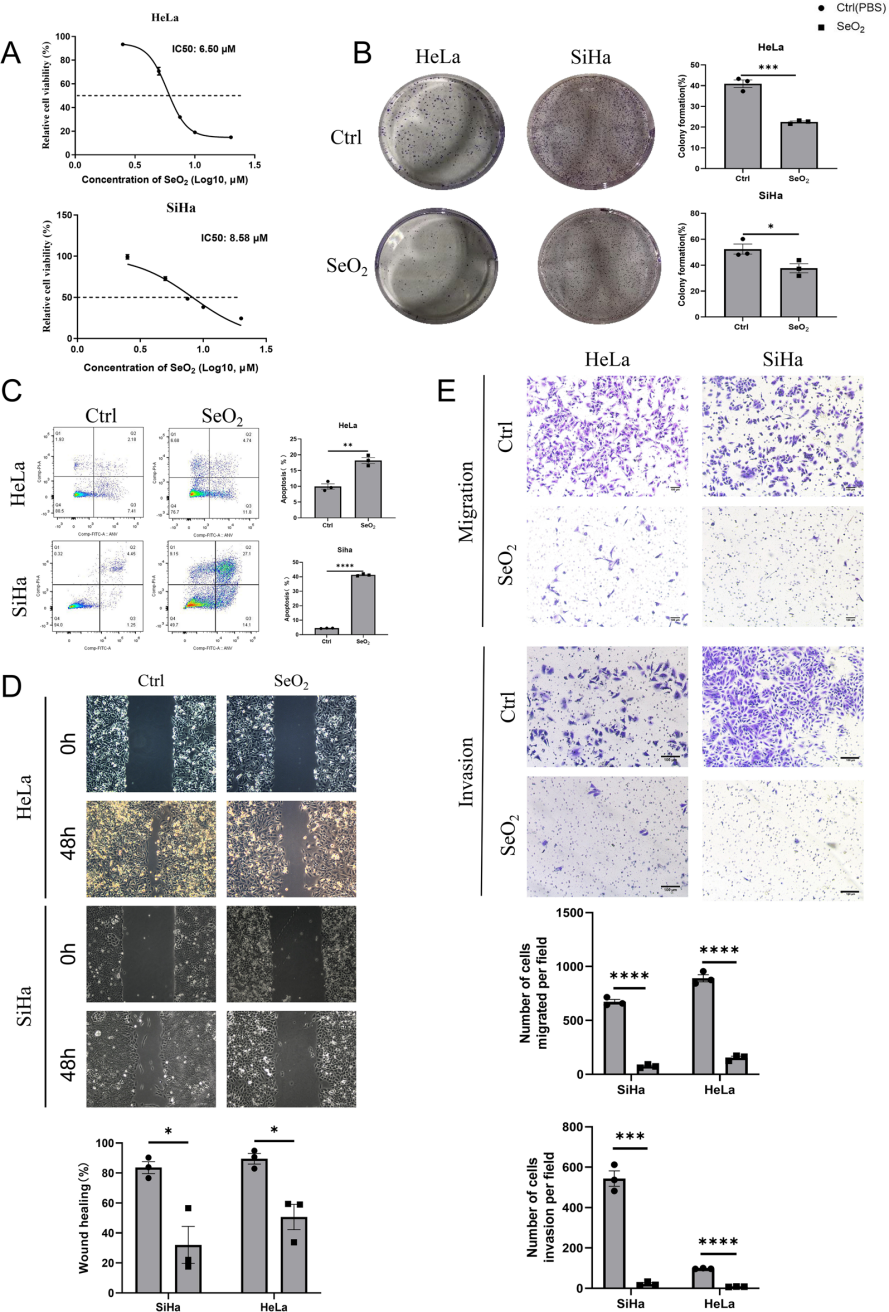
The use of selenium as a treatment demonstrated significant inhibition of various biological behaviors in cervical cancer cells. The impact on cell proliferation and clonogenic ability was evaluated using the CCK-8 assay (**A**) and colony formation assay (**B**) in HeLa and SiHa cells. **C** Apoptosis induction was observed in both cell lines through flow cytometric analysis. **D** The effects of SeO_2_ treatment on cell migration and invasion were assessed using the wound healing assay (scale bar = 100 μm) and **E** Transwell chamber experiments (scale bar = 100 μm) in HeLa and SiHa cells. SeO_2_ treatment led to a significant decrease in cell migration and invasion abilities in both cell lines (*n* = 3). *p < 0.05; **p < 0.01; ***p < 0.001; ****p < 0.0001 compared with PBS-treated Ctrl

We evaluated the effects of SeO_2_ on the proliferation of cervical cancer cells using the CCK-8 Kit. After 24 h of treatment with SeO_2_, significant inhibitory effects on cell proliferation were observed in two cell lines, namely HeLa and SiHa, with IC50 values of 6.50 and 8.58 μM, respectively (Fig. 1A). We also assessed the effect of SeO_2_ on the clonogenic ability of HeLa and SiHa cells using cell plate cloning assays. SeO_2_ at 6.5 and 8.85 μM significantly reduced the clonogenic ability of HeLa and SiHa, respectively, when compared to their untreated controls, both *p* < 0.05 (Fig. 1B). Additionally, flow cytometry revealed that SeO_2_ treatment induced significant apoptosis in the cervical cancer cells after 24 h compared to their PBS-treated controls, respectively, (*p* < 0.05) (Fig. 1C).

Migration and invasion are critical processes in cancer metastasis. To further explore the role of SeO_2_ in cervical cancer cell metastasis, we assessed its effects on cell migration and invasion using scratch healing and Transwell chamber assays. The data showed that SeO_2_ treatment significantly delayed monolayer healing of HeLa and SiHa cells (*p* < 0.05) (Fig. 1D). Similarly, Transwell chamber assay results showed that SeO_2_ treatment significantly decreased the migration and invasion abilities of both cell lines (*p* < 0.05) (Fig. 1E).

### Intraperitoneal administration of selenium effectively suppressed the growth of subcutaneously transplanted cervical cancer tumors

**Fig. 2:**
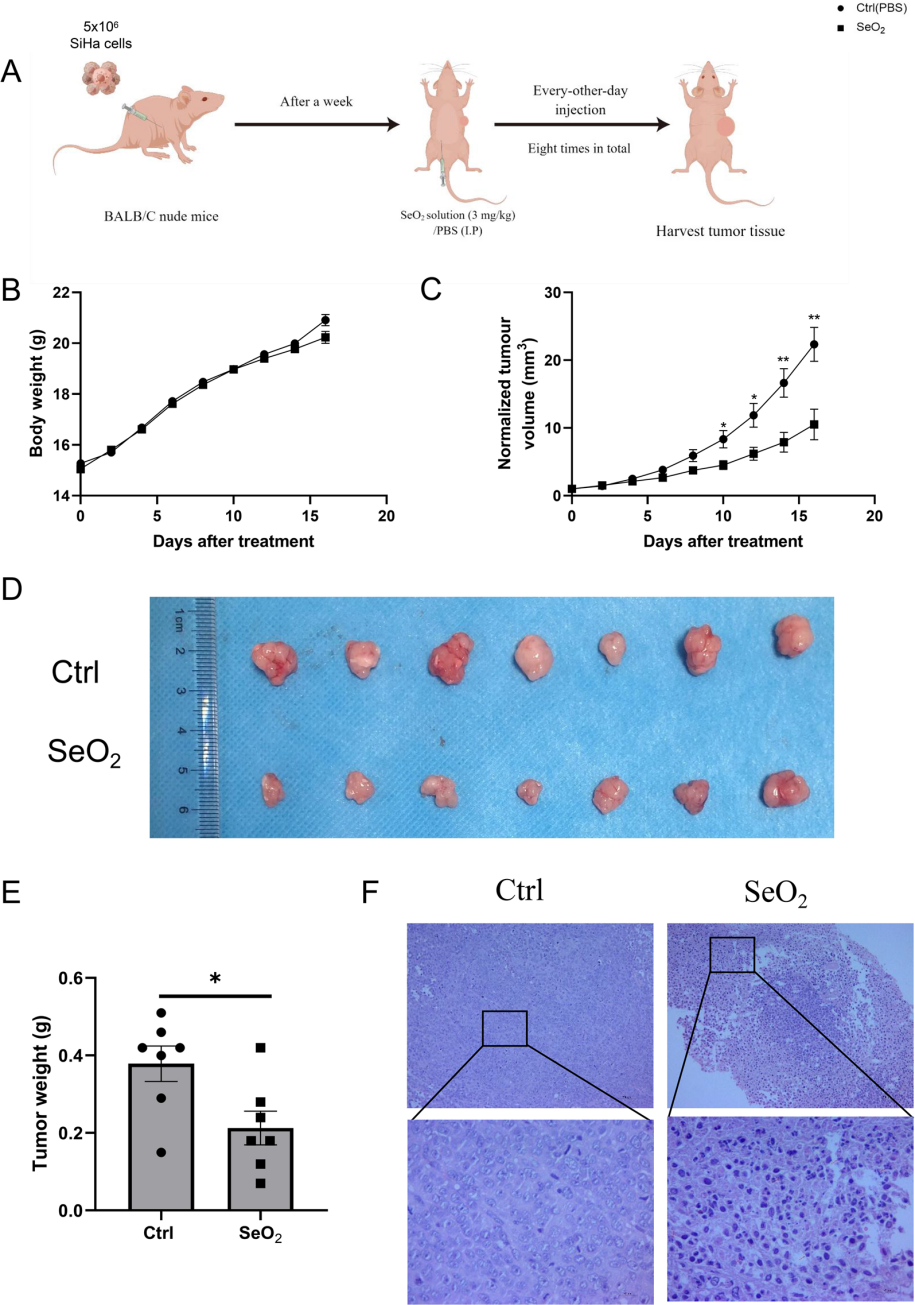
The significant inhibitory effect of selenium on tumor growth in vivo. **A** The experimental scheme for the in vivo studies. **B** The effects of SeO_2_ on the body weight of the inoculated nude mice Tumors from mice received different treatments. The tumor volume and weight were notably reduced by SeO_2_ treatment, as demonstrated in panels **C**, **D**, and **E**. **F** HE staining of the tumor tissues in the SeO_2_ group revealed tumor cells undergoing necrosis, apoptosis, nuclear fragmentation, and dissolution, (*n* = 7). The images were captured at 10 × magnification, with a scale bar of 200 μm, and at 40 × magnification, with a scale bar of 50 μm. *p < 0.05; **p < 0.01 compared with Ctrl (PBS treatment)

To examine the impact of selenium on the growth of cervical cancer tumors in vivo, SiHa cells were injected into the armpit of nude mice. Seven days following the cell injection, SeO_2_ was administered through intraperitoneal injection (Fig. 2A, B). We evaluated the safety and efficacy of SeO_2_ by measuring body weight and tumor volume of the different treatment groups. The results showed that there were no significant changes in body weight after treatment between SeO_2_ treated group and PBS treated group (Fig. 2C). Notably, tumor volume and weight were significantly reduced in the SeO_2_ group compared to the PBS-treated control group (*p* < 0.05) (Fig. 2D, E). Histological HE staining analysis demonstrated that SiHa cells developed poorly differentiated tumors in nude mice. Furthermore, compared to the control group, the SeO_2_ treatment resulted in a higher percentage of tumor cells exhibiting nuclear fragmentation and necrosis (Fig. 2F). These findings indicate that SeO_2_ is an effective growth inhibitor of cervical cancer cells, and its ability to inhibit cell proliferation, migration, and invasion in vitro can be due to its induction of cell apoptosis.

### Selenium exhibited an inhibitory effect on JMJD3 and UTX histone demethylases both in vitro and in vivo

**Fig. 3:**
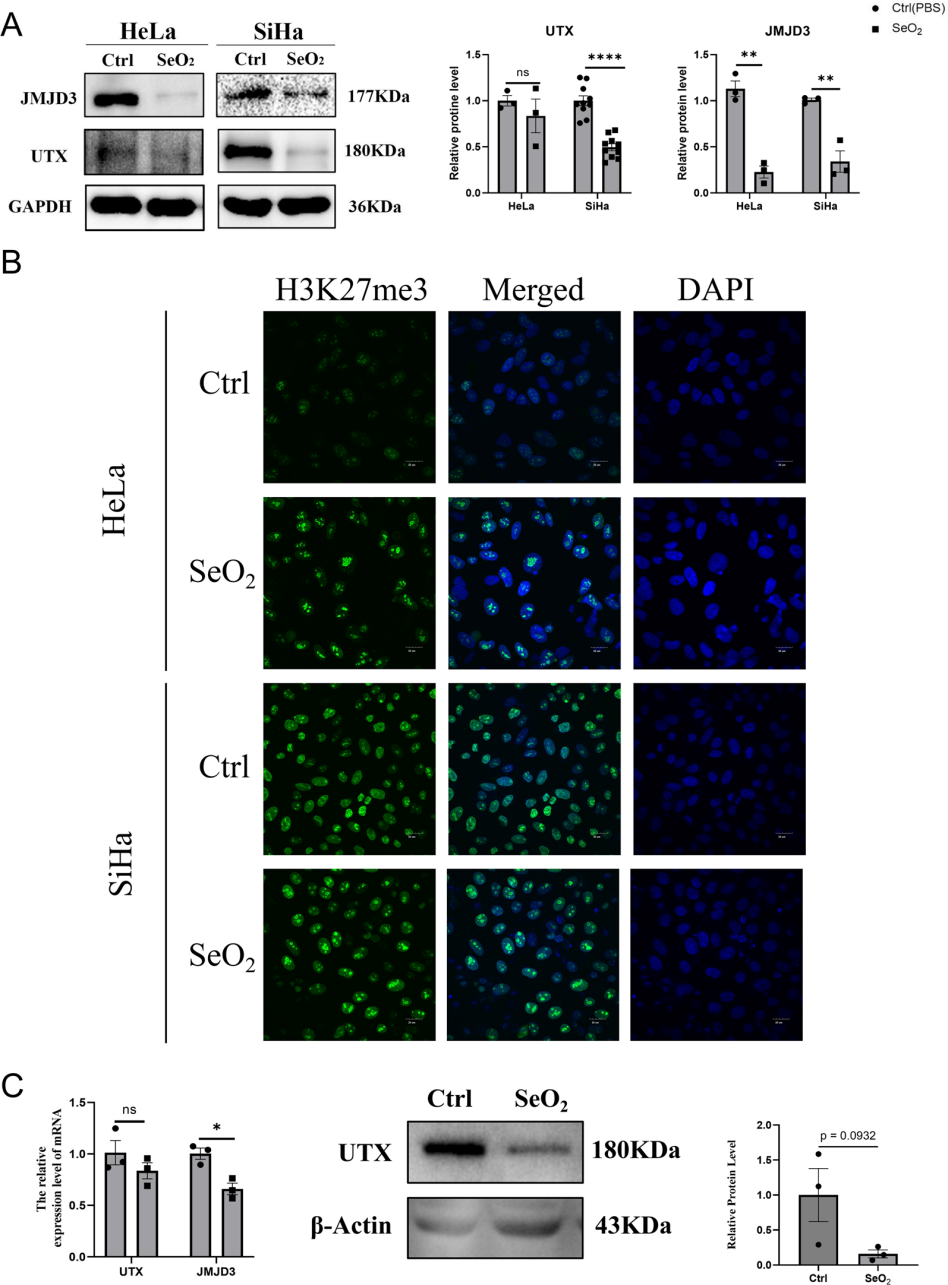
The inhibitory effect of selenium on histone demethylases in cervical cancer. **A** Selenium treatment led to a reduction in the expression of JMJD3 and UTX in HeLa and SiHa cells. **B** An increase in H3K27me3, a marker of histone methylation, in the nuclei of HeLa and SiHa cells following selenium treatment. (Scale bar = 20 μm). **C** Selenium induced a decrease in the expression of JMJD3 and UTX in subcutaneous xenograft tumors of cervical cancer (*n* = 3). *p < 0.05; **p < 0.01; ****p < 0.0001, ns* p* > 0.05 compared with Ctrl

Epigenetic dysregulation is a common feature of most human cancers [25]. Histone methylation plays a crucial role in cancer initiation and development and has thus become a target for pharmacological cancer therapies [26]. Previous studies have shown that selenium can modify epigenetic markers [27]. Therefore, we investigated the effects of SeO_2_ on the expression levels of JMJD3 and UTX in cervical cancer cells and tumor tissues using RT-qPCR and WB analysis. Our Western blot analysis demonstrated that SeO_2_ treatment resulted in a decrease in JMJD3 protein expression in both cell lines (Fig. 3A). Furthermore, UTX was highly expressed in SiHa cells but very low in HeLa cells, and SeO_2_ treatment significantly reduced its expression in SiHa cells. Our findings also demonstrated a significant upregulation of H3K27me3 expression in these cervical cancer cells (Fig. 3B). In addition, the expression level of H3K27me1 was reduced, while H3K27me2 expression was elevated (Additional file 2: Figure S1). In the in vivo experiment, we observed a downregulation of JMJD3 mRNA expression. Although the gene level of UTX was not altered with SeO_2_ administration, we observed a significant reduction in UTX protein expression upon SeO_2_ treatment (Fig. 3C). These results suggest the potential of selenium as a therapeutic approach for cervical cancer treatment in vivo.

### Inhibition of histone H3K27 demethylases impaired cervical cancer cell growth and induced apoptosis, potentially through the downregulation of cell cycle and apoptosis-related genes

**Fig. 4:**
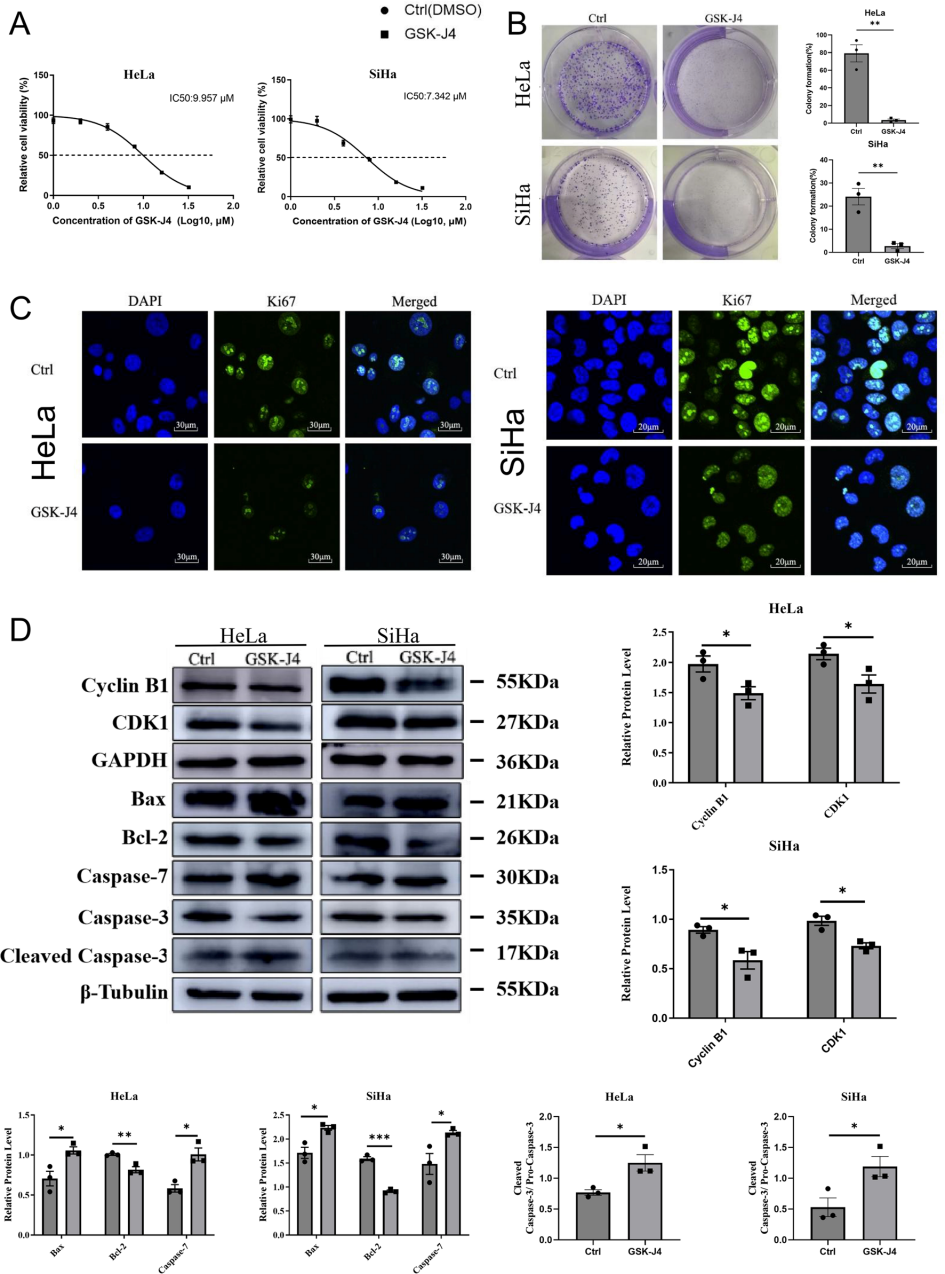
The effects of inhibiting histone H3K27 demethylases on the proliferation of cervical cancer cells. **A** The inhibitory effect of GSK-J4 on cell proliferation through the use of the CCK-8 Kit. **B** The results of colony formation assay in HeLa and SiHa cells treated with GSK-J4. **C** The immunofluorescence staining of Ki67, a marker of cell proliferation, using CLSM. The left image represents HeLa cells and the right image represents SiHa cells. The scale bar in the left image is 30 μm, and in the right image is 20 μm. **D** The results of Western blot analysis for Cyclin B1, CDK1, Bcl-2, Bax, caspase-7, caspase-3, and cleaved caspase-3 (*n* = 3). *p < 0.05; **p < 0.01; ***p < 0.001; and ****p < 0.0001 compared to the control group

To investigate the role of JMJD3 and UTX in cervical cancer, we utilized GSK-J4, a specific inhibitor of histone H3K27 demethylase. Cell viability was assessed using the CCK-8 Kit to evaluate the impact of histone H3K27 demethylase inhibition on cell proliferation. Our results showed significant inhibitory effects on HeLa and SiHa cells, with IC50 values of 9.957 μM and 7.342 μM, respectively. For all subsequent experiments, we used a GSK-J4 concentration of 8 μM unless otherwise specified. A colony formation assay was performed to assess the effect of histone H3K27 demethylase inhibition on cell growth, and after 7 days of treatment, the colony formation ability of cervical cancer cells was significantly impaired (*p* < 0.05) (Fig. 4B).

To further explore the effect of GSK-J4 on cervical cancer cells, we used flow cytometry to analyze cell cycle distribution and apoptosis. After 72 h of GSK-J4 treatment, the percentage in the G2/M phase of HeLa and SiHa cells increased significantly, while the percentage of cells in the G0/G1 phase decreased significantly compared to the control group (*p* < 0.05, Additional file 3: Figure S2A). Additionally, treatment with GSK-J4 led to increased apoptosis in both cell lines (*p* < 0.05) (Additional file 3: Figure S2B). We then performed qRT-PCR and Western blot analysis to evaluate the effect of histone demethylase H3K27 inhibition on cell cycle and apoptosis-related genes. Our results showed that the expression levels of Cyclin B1, CDK1, Bcl-2, and Ki67 were significantly downregulated in both HeLa and SiHa cells after histone demethylase inhibition (Fig. 4D; Additional file 3: Figure S2C). Furthermore, the expression of P16INK4A mRNA was notably downregulated in SiHa cells and slightly decreased in HeLa cells (Additional file 3: Figure S2C). In contrast, the expression levels of Bax, caspase-7, and cleaved caspase-3 were significantly increased in both HeLa and SiHa cells after histone demethylase inhibition (*p* < 0.05) (Fig. 4D).

### Inhibition of histone H3K27 demethylase can effectively suppress the invasive and migratory potential of cervical cancer cells

**Fig. 5:**
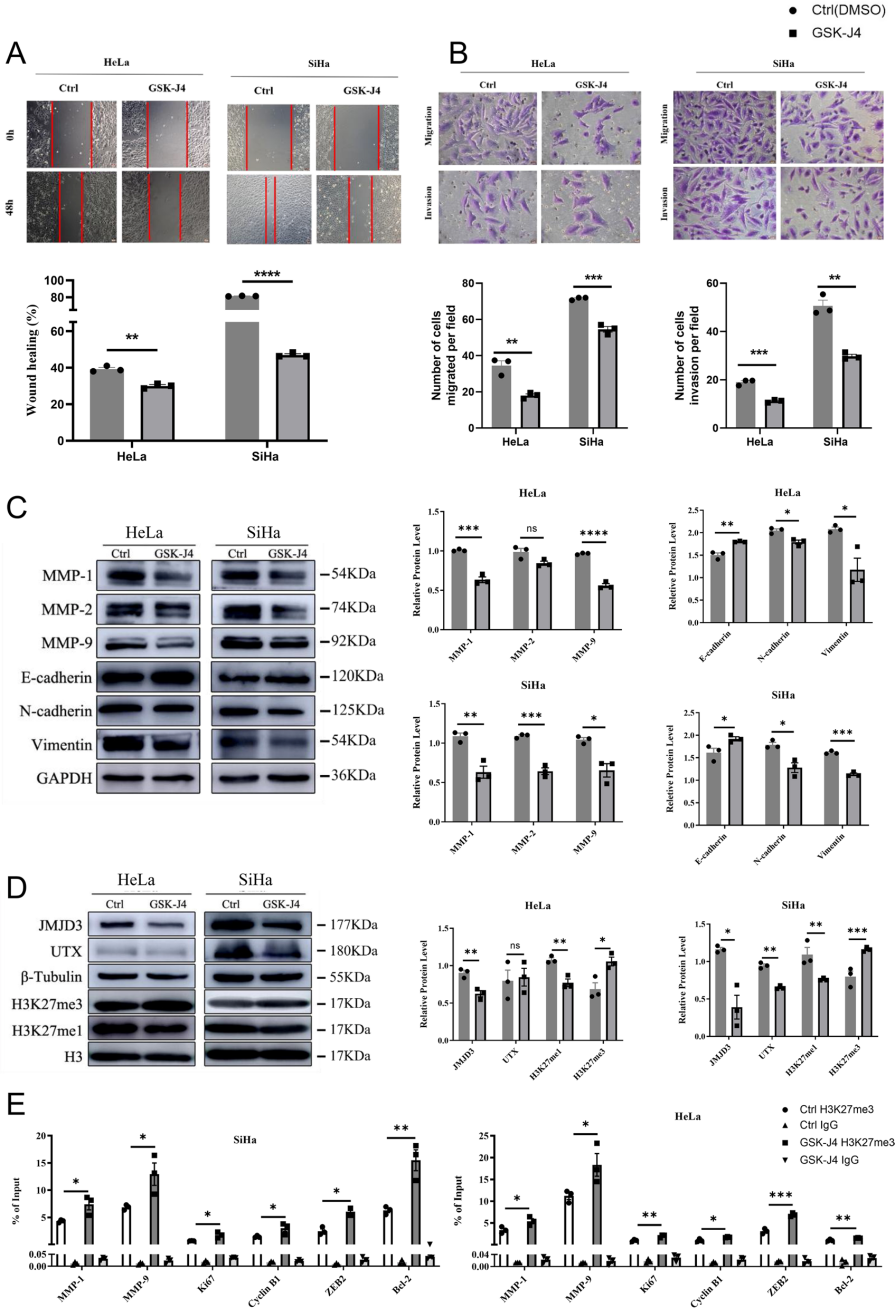
The impact of histone demethylase inhibition on the migration, invasive potential, and epithelial-mesenchymal transition of cervical cancer cells was studied. **A** The results of the wound healing assay, which measures cell migration. The scale bar is 50 μm, and **B** the outcomes of the Transwell chamber experiments, which assess cell invasion. The scale bar is 12.5 μm. **C** The results of Western blot analysis, evaluating the effects of histone demethylase inhibition on cell migration, invasion, and the expression of EMT-related proteins. **D** The impact of histone demethylase inhibition on global H3K27me3 and H3K27me1 in HeLa and SiHa cells, as determined by Western blot. **E** The changes in the enrichment of H3K27me3 binding to the promoter regions of target genes were determined using ChIP-qPCR (*n* = 3). *p < 0.05; **p < 0.01; ***p < 0.001; ****p < 0.0001, compared to DMSO Ctrl

Our study revealed that 48-h GSK-J4 treatment significantly delayed the wound healing ability of the cervical cancer cells (*p* < 0.05, Fig. 5A) and suppressed their migration and invasion abilities by Transwell chamber assays (*p* < 0.05, Fig. 5B). Moreover, the mRNA levels of MMP-1 and MMP-9 in HeLa cells and MMP-2 and MMP-9 in SiHa cells decreased significantly with the treatment of GSK-J4 (*p* < 0.05, Fig. 5C). We also observed a significant downregulation of EMT-related markers, including CDH2, VIM, ZEB1, and ZEB2, and upregulation of CDH1 in both cell types (*p* < 0.05, Fig. 5C). These findings were confirmed by western blot analysis, which showed a reduction in MMP-1 protein levels in SiHa cells after GSK-J4 treatment (*p* < 0.05, Fig. 5C). Overall, our results suggest that inhibiting histone demethylase could suppress MMP expression, reverse EMT of cervical cancer cells.

Furthermore, we observed a significant increase in global H3K27me3 in HeLa and SiHa cells after inhibiting histone demethylase (Fig. 5D). To investigate how H3K27me3 regulates proliferation-, migration-, and EMT-related genes, we performed ChIP-qPCR and found a remarkable recruitment of H3K27me3 around the TSS regions of Ki67, Cyclin B1, Bcl-2, MMP-1, and MMP-9 in HeLa and SiHa cells, respectively (*p* < 0.05) (Fig. 5E). These results suggest that specific inhibition of histone demethylase may upregulate H3K27me3 level and epigenetically impair the expression of genes related to cell survival, migration, and EMT, leading to the observed anti-tumor effects.

### Inhibition of histone H3K27 demethylases suppressed the growth of human cervical cancer cells in nude mice model

**Fig. 6:**
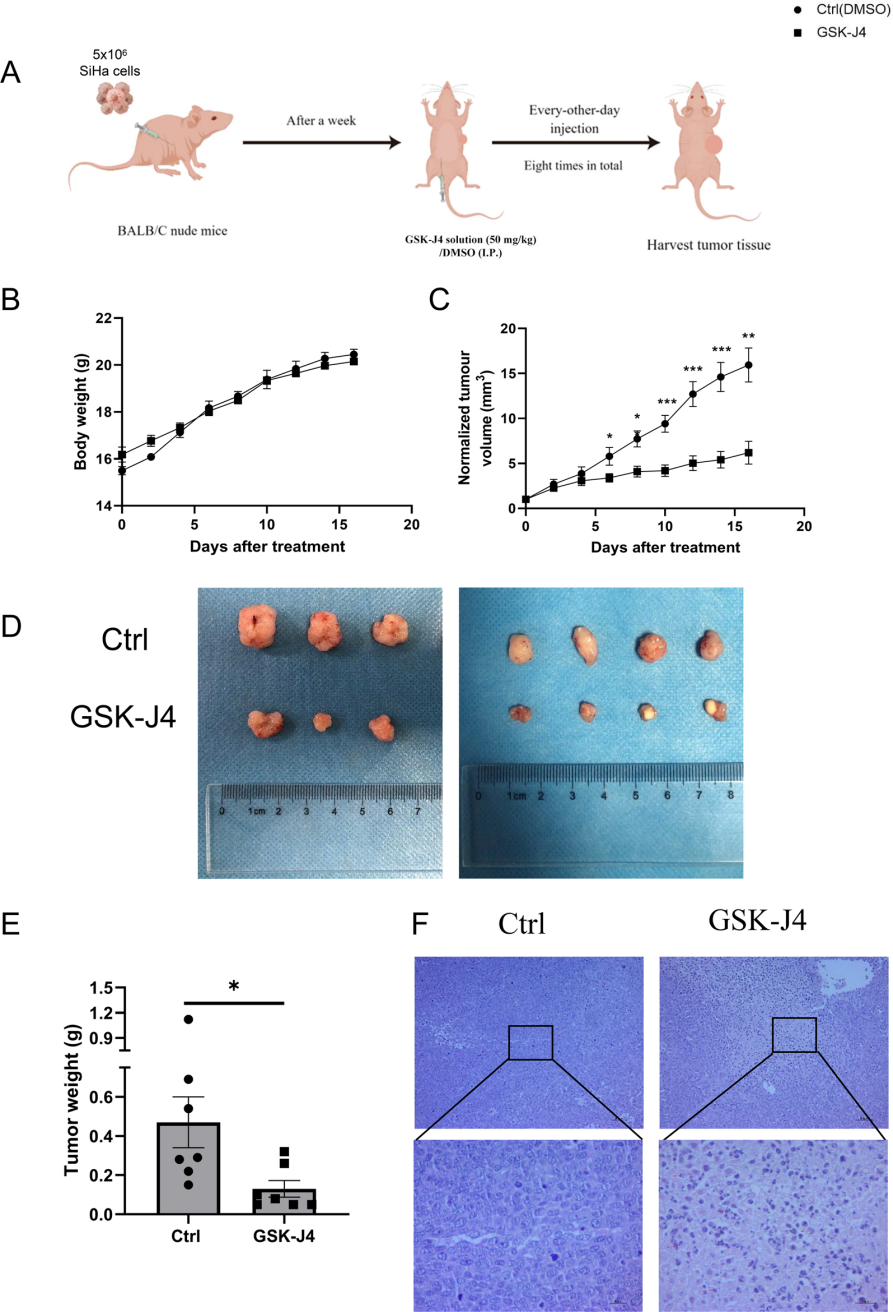
The significant inhibition of tumor growth in vivo by the histone demethylase inhibitor GSK-J4. **A** The experimental design for the in vivo experiments. **B** The effect of GSK-J4 on the body weight of nude mice. **C**), **D**, and **E** demonstrate the impact of GSK-J4 on tumor volume and tumor weight, respectively. **F** the results of HE staining of tumor tissues subjected to different treatments. The images at 10 × magnification have a scale bar of 200 μm (upper), while the images at 40 × magnification have a scale bar of 50 μm (lower), (*n* = 7). *p < 0.05; **p < 0.01; ***p < 0.001 compared with DMSO Ctrl

To investigate the role of JMJD3 and UTX in cervical cancer growth, we established an in vivo model by subcutaneously implanting SiHa cells in female 4-week-old athymic nude mice to form xenografts. Seven days after SiHa inoculation, the mice were subsequently administered GSK-J4, the inhibitor targeting JMJD3 and UTX. A total of eight intraperitoneal administrations of 50 mg/kg of GSK-J4 were conducted, with each session administered once every 2 days (Fig. 6A). There was no significant change in body weight observed during the treatment when compared to the control group treated with DMSO (Fig. 6C). However, treatment with GSK-J4 significantly inhibited the growth rate and reduced the weight of the xenografts compared to the control group (Fig. 6B, D, E). HE staining also showed significant nuclear fragments in cervical cancer cells after GSK-J4 treatment (Fig. 6F). These findings suggest that the inhibition of histone demethylases effectively suppresses cervical cancer cells in vivo.

### Validation of the clinical relevance of JMJD3 and UTX expression in cervical cancer and inhibition of cervical carcinoid tumors by selenium and H3K27 demethylase inhibitors

**Fig. 7:**
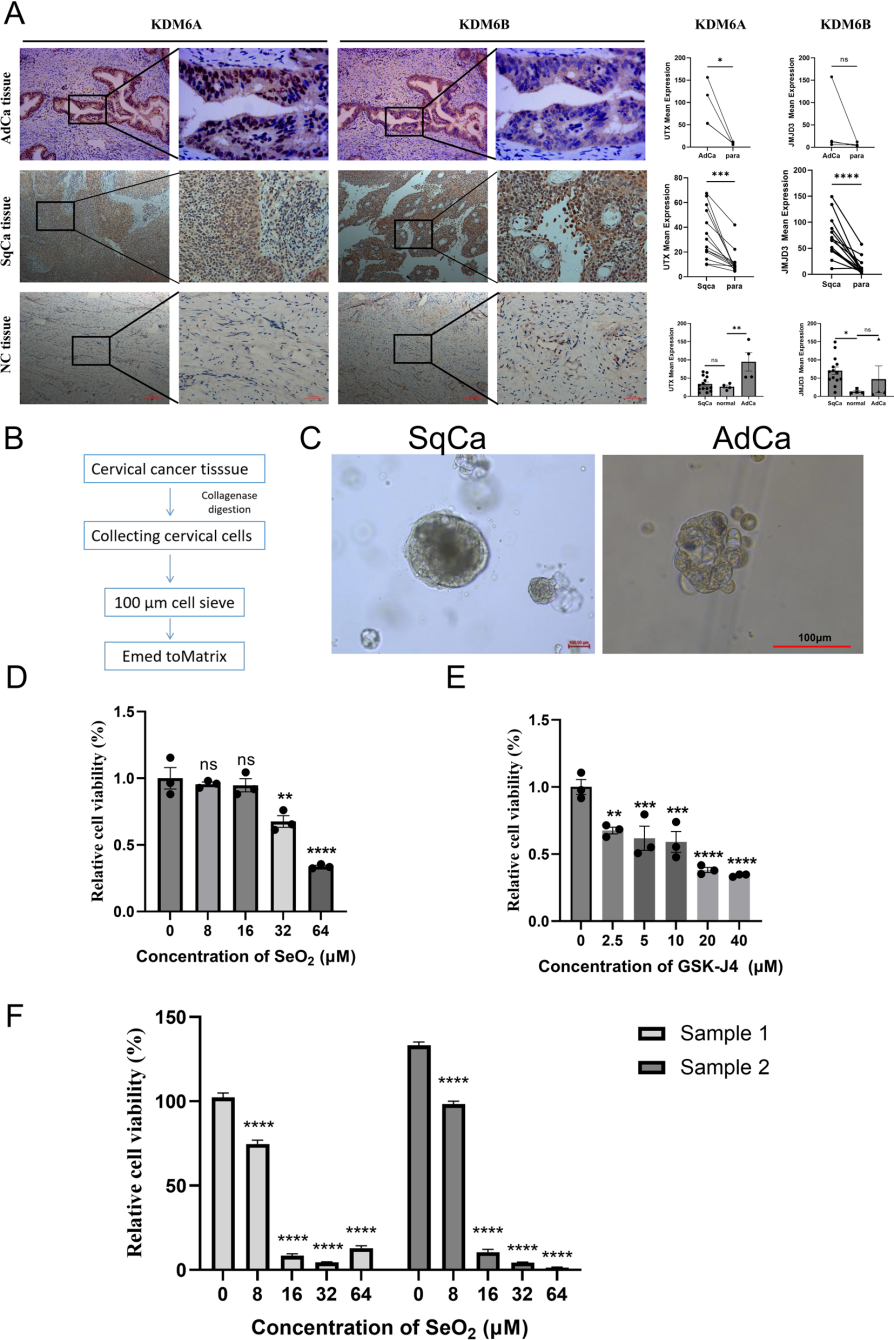
The establishment of cervical cancer organoids and their response to the treatment of selenium and GSK-J4. **A** The immunohistochemical analysis evaluating the expression of histone demethylases UTX (KDM6A) and JMJD3 (KDM6B) in cervical cancer and normal cervical tissues. The left column displays images taken under 10 × magnification, with a scale bar of 200 μm, while the right column shows images taken under 40 × magnification, with a scale bar of 50 μm. **B** A schematic of cervical cancer organoids generating. **C** shows representative images of cervical cancer organoids. **D** The effects of different concentrations of selenium dioxide and **E** GSK-J4 on the viability of cervical cancer organoids, as assessed using the CCK8 assay. **F** The impact of different concentrations of selenium dioxide on the viability of three-dimensional bio-printed cervical cancer models. **p < 0.01; ***p < 0.001; ****p < 0.0001; ns *p* > 0.05

To reinforce the clinical implications of our findings, we obtained cervical cancer samples to confirm the expression of UTX (KDM6A) and JMJD3 (KDM6B). Immunohistochemical staining was used to assess the expression of UTX and JMJD3 in paired cervical cancer tissues, and corresponding adjacent normal tissues, as well as non-cancer normal tissues. Our findings reveal that the expression of UTX was significantly elevated in both cervical squamous cell carcinoma tissues and adenocarcinoma compared to their corresponding adjacent normal tissues. Only adenocarcinoma exhibited a significant increase in UTX expression when compared to normal cervical tissues, while the increase in cervical squamous cell carcinoma was not statistically significant. Additionally, JMJD3 expression in cervical squamous cell carcinoma tissues was significantly higher than in their corresponding adjacent normal tissues and non-cancer normal tissues (Fig. 7A). This highlights the potential usefulness of histone demethylases as diagnostic or prognostic markers in cervical cancer.

The use of patient-derived tumor organoids provides an ideal platform for studying personalized drug responses in vitro, as they preserve the genetic heterogeneity of the primary tumor [28]. Organoid culture from specific individuals has the potential to become a powerful tool for precision therapy. Surgically resected fresh cervical cancer samples were inoculated into cervical organoid culture-medium, resulting in the establishment of organoid lines from patients (Fig. 7B). The derived tumor organoids displayed different morphologies, ranging from dense to cystic (Fig. 7C; Additional file 4: Figure S3A). To evaluate whether organoids accurately recapitulate the heterogeneity observed in cervical cancer tumors, we performed HE staining on three selected organoids and their corresponding original tissues. The results demonstrated similar histological patterns between the organoids and patient samples, with both showing invasive squamous cell carcinoma characteristics that were preserved during culturing (Additional file 4: Figure S3A). Additionally, STR analysis was conducted on two constructed organoids and their original tissues, revealing a 100% match and confirming their identical origin without any presence of multiple alleles (Additional file 4: Figure S3B). Furthermore, genotyping results showed inconsistency between tested samples and positive controls, validating the genetic stability of these patient-derived organoids (Additional file 4: Figure S3C). Subsequently, we investigated whether selenium and GSK-J4 could effectively suppress cervical cancer patient-derived organoids by conducting CCK-8 assays to measure their activity after treatment with varying concentrations of selenium and GSK-J4 over 3 days. The results indicated a dose-dependent inhibition of cervical cancer organoids by specific H3K27 demethylase inhibitor, GSK-J4 (Fig. 7D, E). Similarly, we found that selenium significantly promoted apoptosis in HCCOs (Additional file 5: Figure S4) as well as inhibited cervical cancer models constructed by 3D printing (Fig. 7F).

## Discussion

Selenium's complex association with the development of cancer has drawn significant attention, sparking a growing interest in research [20, 21, 29]. The promising prospect of selenium supplementation as a risk-reducing strategy for various cancers has ignited hope in the medical community [30, 31]. Selenium compounds have relatively low preparation costs and lower prices, which enables them to have a wider potential application in the field of therapy. Delving deeper into the molecular realm, the dynamic landscape of epigenetic modifications emerges as a pivotal player in governing tumor progression [32, 33]. Excitingly, emerging evidence suggests that selenium can orchestrate apoptosis through intricate epigenetic mechanisms, unveiling novel therapeutic avenues [27, 34, 35]. However, within the domain of cervical cancer, the enigmatic role of selenium and its interplay with epigenetics await comprehensive elucidation, beckoning further investigation into this captivating scientific frontier.

Our research has highlighted the potential of selenium as a therapeutic solution for cervical cancer. We observed that selenium effectively suppressed the growth, migration, and invasion of cervical cancer cells while also promoting apoptosis. In vivo, studies involving intraperitoneal selenium dioxide solution demonstrated its impressive ability to inhibit cancer cell proliferation after the tumor had been established for 7 days. Additionally, treated mice did not show any significant changes in body weight, indicating the safety and efficacy of selenium as a therapeutic agent for cervical cancer. Furthermore, we generated HCCOs from resected clinical tissue and discovered that selenium exhibited potential in treating human cervical cancer. These results provide insight into the promising role of selenium in cancer treatment and pave the way for future investigations into its mechanisms of action and clinical applications.

Within the chromatin structure, histones are important proteins regulating gene expression through modifications [36]. Histone H3K27 trimethylation (H3K27me3) is an important inhibitory modification, and its regulation of gene expression mainly depends on two histone demethylases: JMJD3 and UTX. These two enzymes can remove the trimethylation modification of histone H3K27, thereby making chromatin more open and allowing gene expression to be activated [37, 38]. The results of our study indicate that GSK-J4, a specific histone H3K27 demethylase inhibitor, shows promise as a therapeutic approach for treating cervical cancer. Previous research has shown that JMJD3 and UTX have oncogenic properties in various malignancies [39, 40]. The deregulation of H3K27me3 can lead to the abnormal activation of cancer-related genes [41]. Inhibiting H3K27me3 demethylation has demonstrated promising anticancer effects in breast cancer [42], lung cancer [43], and colorectal cancer [44].

Furthermore, our study demonstrated that histone demethylases, specifically JMJD3 and UTX, are highly expressed in cervical cancer cells and their expression can be suppressed by selenium. This suggests that the anti-tumor effects of selenium on cervical cancer cells may be attributed to its regulation of histone demethylation. Additionally, our findings provide important insights into the underlying mechanisms through which selenium influences cervical cancer cells, both in laboratory settings and in living organisms. Moreover, we found that specific inhibition of histone H3K27 demethylases with GSK-J4 produced similar effects. This included reduced viability, colony formation, invasive and migratory properties of cervical carcinoma cells in vitro, as well as induced G2/M phase arrest and facilitated cell apoptosis. Additionally, inhibiting histone demethylases reversed epithelial-mesenchymal transition (EMT), a key process implicated in cancer metastasis [45–47]. Furthermore, the administration of GSK-J4, the histone demethylase inhibitor, effectively suppressed the growth of subcutaneous cervical cancer xenografts established for 7 days and hindered the progression of HCCOs.

We then performed ChIP-qPCR analysis and observed a significant increase in H3K27me3 enrichment around the TSS regions of key genes involved in cell proliferation, migration, and EMT in HeLa and SiHa cells following histone demethylase inhibition. These genes, including Ki67, Cyclin B1, Bcl-2, MMP-1, and MMP-9, exhibited a statistically significant difference with *p* < 0.05. Our findings suggest that inhibiting histone demethylase leads to an upregulation of H3K27me3, resulting in the epigenetic suppression of genes crucial for tumor cell survival, migration, and EMT. This mechanism ultimately contributes to the profound anti-tumor effects observed in our study.

It is worth mentioning that JMJD3 and UTX, besides their roles as H3K27 demethylases, have been found to independently regulate gene expression [48]. Selenium inhibits the expression of JMJD3 and UTX, preventing their demethylation of histone H3K27 and resulting in more condensed chromatin and suppressed gene expression. On the other hand, GSK-J4 directly inhibits the enzymatic activity of JMJD3 and UTX, leading to an increased trimethylation modification of histone H3K27, compacting chromatin, and suppressing gene expression [49, 50]. It is important to note that while both selenium and GSK-J4 affect histone demethylase function, their mechanisms of action differ. Although both have demonstrated promising safety profiles, further research is required to fully understand the anti-tumor and gene expression regulatory effects of selenium and GSK-J4, as their spectra of activity may not be entirely consistent.

To summarize, our study underscores the promise of selenium as a potential therapeutic agent for cervical cancer. By inhibiting histone demethylase activity, selenium effectively suppresses cell proliferation, migration, and invasion. Our results also demonstrate that inhibiting histone demethylase activity hinders the growth of human cervical cancer organoids. The in vivo findings support the safety and efficacy of selenium, and the histone demethylase inhibitor GSK-J4 holds significant potential in clinical applications. These findings deepen our understanding of selenium's role in cancer treatment and provide a foundation for future research in this field.

### Supplementary Information

**Additional file 1: Table S1**. Primers used for the qPCR assay; **Table S2**. Primers used for the ChIP-qPCR assay.

**Additional file 2: Figure S1**. Selenium dioxide upregulates H3K27me2 expression and decreases H3K27me1 expression.

**Additional file 3: Figure S2.** Effect of inhibition of histone demethylases on cell cycle distribution (A) and apoptosis (B) in cervical cancer cells assessed by flow cytometry. (C, D) Effects of histone demethylase inhibition on the proliferation, cell cycle, migration, invasion, and epithelial-mesenchymal transition related genes of cervical cancer cells. *p < 0.05, **p < 0.01, ***p < 0.001, ****p < 0.0001, ns p > 0.05.

**Additional file 4: Figure S3.** (A) Hematoxylin and eosin (H&E) of paraffin-embedded cervical cancer tissue (Scale bars = 200 μm) and corresponding organoids (Scale bars = 5 μm). STR identification (B) and positive control (C) of cervical cancer original tissues and organoids.

**Additional file 5: Figure S4.** Live/Dead staining of SeO_2_-treated HCCOs. Viable cells were labeled green and dead cells were labeled red. Scale bar = 50 μm.

## Data Availability

The data sets used and/or analyzed during the current study are available from the corresponding author on reasonable request.

## Abbreviations

AdCa: Adenocarcinoma
Bax: Bcl-2 associated X protein
Bcl-2: B-cell lymphoma/Leukemia-2
CDH2: Cadherin 2
CDK1: Cyclin-dependent kinase 1
ChIP-qPCR: Chromatin immunoprecipitation coupled with quantitative PCR
DMSO: Dimethyl sulfoxide
EMT: Epithelial-mesenchymal transition
GAPDH: Glyceraldehyde-3-phosphate dehydrogenase
H3K27me1: Monomethylation at lysine 27 of histone H3
H3K27me2: Dimethylation at lysine 27 of histone H3
H3K27me3: Trimethylation at lysine 27 of histone H3
HCCOs: Human cervical cancer-derived organoids
HE staining: Hematoxylin–eosin staining
HPV: Human papillomavirus
IC50: Half-maximal inhibitory concentration
IHC: Immunohistochemistry
UTX (KDM6A): Lysine (K)-specific demethylase 6A
JMJD3 (KDM6B): Lysine (K)-specific demethylase 6B
Ki67: Marker of proliferation Ki67
MMP-1: Matrix metalloproteinase-1
MMP-2: Matrix metalloproteinase-2
MMP-9: Matrix metalloproteinase-9
NF-kappa B: Nuclear factor-kappa B
PBS: Phosphate-buffered saline
qRT-PCR: Reverse transcription-quantitative polymerase chain reaction
ROS: Reactive oxygen species
SEM: Standard error
SeO_2_: Selenium dioxide
SqCa: Squamous cell carcinoma
TSS: Transcription start site
VIM: Vimentin
ZEB1: Zinc finger E-box binding homeobox 1
ZEB2: Zinc finger E-box binding homeobox 2
