# Supplementary material for: Targeting histone demethylases JMJD3 and UTX: selenium as a potential therapeutic agent for cervical cancer
Source: Clin Epigenetics. 2024 Apr 4;16:51. doi: 10.1186/s13148-024-01665-3 (PMC10993516; doi:10.1186/s13148-024-01665-3)
Supplement: Supplementary file 1 — Additional file 1: Table S1. Primers used for the qPCR assay; Table S2. Primers used for the ChIP-qPCR assay. [file 13148_2024_1665_MOESM1_ESM.docx]

| **Gene** | **Forward/reverse** | **Primer sequence（5′➝3′）** |
| --- | --- | --- |
| Bax  Bcl-2  Cyclin B1  CDK1  CDH1  CDH2  Ki67  MMP-1  MMP-2  MMP-9  P16INK4A  VIM  ZEB1  ZEB2  GAPDH | F  R  F  R  F  R  F  R  F  R  F  R  F  R  F  R  F  R  F  R  F  R  F  R  F  R  F  R  F  R | CCCGAGAGGTCTTTTTCCGAG  CCAGCCCATGATGGTTCTGAT  GGTGGGGTCATGTGTGTGG  CGGTTCAGGTACTCAGTCATCC  GGGGACCCAAACCTCTGTA  TGCAATAAACATGGCCGTTA  AAACTACAGGTCAAGTGGTAGCC  TCCTGCATAAGCACATCCTGA  CGAGAGCTACACGTTCACGG  GGGTGTCGAGGGAAAAATAGG  TCAGGCGTCTGTAGAGGCTT  ATGCACATCCTTCGATAAGACTG  CAGACCCATTTACTTGTGTTGGA  TATTGGAGCAGCAAGAGGCT  GGCTGCTTCATCACCTTCAG  CTACTGAGTGGCCGTGTTTG  TCCCTGAGGTTCTCTTGCTG  GACAAGCTCTTCGGCTTCTG  CAAAGTTCGAGGTGGTAGCG  GATCCAGGTGGGTAGAAGGTC  CCCCTGCAAACTTCGTCCT  GACGCCATCAACACCGAGTT  CTTTGTCGTTGGTTAGCTGGT  GATGATGAATGCGAGTCAGATGC  ACAGCAGTGTCTTGTTGTTGT  CAAGAGGCGCAAACAAGCC  GGTTGGCAATACCGTCATCC  GGAGCGAGATCCCTCCAAAAT  GGCTGTTGTCATACTTCTCATGG |

**Table S1 The primers used for qPCR assay**

| **Gene** | **Forward/reverse** | **Primer sequence（5′➝3′）** |
| --- | --- | --- |
| Bcl-2  Cyclin B1  Ki67  MMP-1  MMP-9  ZEB2 | F  R  F  R  F  R  F  R  F  R  F  R | GCAGAAGTCTGGGAATCG  GCATAAGGCAACGATCC  CAGAGAGTTGTTGCAACGATCAAA  CACACTTTCCATTTCTACGGTAGC  AATCTTCTGGCAATGAGTAATGT  ATAACCCGTCCTGCTATCC  AGTGTTAGTAATTCCACCCTCTGC  GGTCAAAGAGTACTCCATGGTCTT  AGCACTTGCCTGTCAAGGAG  ATGGTGAGGGCAGAGGTGT  GGGCAGAGAACTTTGTTCCA  GGTGCACACCATTCACAGAA |

**Table S2 The primers used for ChIP-qPCR assay**
